# The Hydrophobic Effect Contributes to the Closed State of a Simplified Ion Channel through a Conserved Hydrophobic Patch at the Pore-Helix Crossing

**DOI:** 10.3389/fphar.2015.00284

**Published:** 2015-11-27

**Authors:** Michael Yonkunas, Maria Kurnikova

**Affiliations:** Department of Chemistry, Carnegie Mellon University, PittsburghPA, USA

**Keywords:** hydrophobic effect, ion channels, glutamate receptor, free energy, channel wetting

## Abstract

Ion selectivity-filter structures are strikingly similar throughout the large family of K^++^ channels and other p-loop-like receptors (i.e., glutamate receptors). At the same time, the triggers for opening these channels, or gating, are diverse. Two questions that remain unanswered regarding these channels are: (1) what force(s) stabilize the closed non-conducting channel-pore conformation? And (2) what is the free energy associated with transitioning from a closed (non-conducting) to an open (conducting) channel-pore conformation? The effects of charge and hydrophobicity on the conformational states of a model tetrameric biological ion channel are shown utilizing the amino acid sequence from the K^+^ channel KcsA as the model “channel”. Its widely conserved hydrophobic bundle crossing located adjacent to the lipid head-groups at the intracellular side of the membrane was calculated to have a 5 kcal/mol free energy difference between modeled open and closed conformations. Simulated mutants of amino acids within the hydrophobic region significantly contribute to the size of this difference. Specifically for KcsA, these residues are part of the pH sensor important for channel gating and our results are in agreement with published electrophysiology data. Our simulations support the idea that the hydrophobic effect contributes significantly to the stability of the closed conformation in tetrameric ion channels with a hydrophobic bundle crossing positioned in proximity to the lipid head groups of the biological membrane.

## Introduction

Cellular membranes use ion channel pores to maintain cellular volume, or regulate ion flux through a process known as gating. These channel pores are often formed as protein complexes that consist of several subunits. A protein quaternary structure formed by complementary surfaces at the subunit interface thus determines a channel structure. The central pore of an open protein channel is typically a hydrophilic water-filled pathway for ion conduction across the highly hydrophobic environment of the cellular membrane. Occasionally these hydrophilic pores contain hydrophobic patches that impede ion flux or increase ion flux [e.g., chloride channels (Kardos, 1993)] by promoting water-less volumes inside otherwise water-filled pores. Such molecular-level drying (Zhu and Hummer, 2012) or phase behavior of water (Roth et al., 2008) have been implicated as an effective block to ion conduction in proteins. Theoretical studies and molecular dynamics (MDs) simulations indicate that hydrophobic surfaces within channel lining helices create a block to ion flux (Kardos, 1993; Hummer et al., 2001; Beckstein and Sansom, 2003, 2006; Anishkin and Sukharev, 2004; Sotomayor and Schulten, 2004; Liu et al., 2005; Roth et al., 2008; Cheng et al., 2009; Jensen et al., 2010; Nury et al., 2010; Zhu and Hummer, 2010, 2012). But in order to modulate the phase of pore water and thus impede conduction (Chandler, 2005) an ion channel must be able to modulate the geometry of the channel pore. Changes in the quaternary structure of an ion channel have also been suggested as a direct physical gate to ion flux (Miloshevsky and Jordan, 2007). In view of such variety of ion regulation mechanisms by ion channels, it then becomes important to understand the energetic relationships between various conformations of essentially protein *complexes* during gating and their implications for ion conductance.

For transmembrane proteins and ion channels in general, a hydrophobic patch within a transmembrane helix that forms the channel pore may play many different roles for the pore-lining helices of an ion channel. For example, the patch can stabilize the pore by forming a network of hydrogen bonds between the patch residues and the surrounding (non-pore-lining) transmembrane helices thus reducing the free energy cost of being in the bilayer (Li et al., 2012; Lin et al., 2012). In this case the hydrophobic patch is preferably located deep in the hydrocarbon tails of the lipid bilayer, an ideal environment for stabilizing the hydrogen-bonded backbone of ion channel helical transmembrane regions (White and Wimley, 1999). It is because of this non-polar environment that the patch will not exhibit strong interactions with itself (i.e., it is not a “sticky patch” in the channel pore). The mechanosensitive channels McsL and MscS contain such regions that the protein uses as a hydrophobic gate, stabilized by protein hydrogen bonds, and manipulated by voltage and tensile forces of the membrane (Anishkin et al., 2010). Alternatively, a hydrophobic patch can be located more proximal to the lipid head groups of the membrane. In the voltage gated *Shaker* and KvaP K^+^ channels, a small patch of hydrophobic residues on the S4 helices has been recognized as being important for stabilizing the channel in the active or inactive conducting state (Xu et al., 2010; Zheng et al., 2011). In a similar fashion, a patch of helix containing a larger number of hydrophobic residues such as the intracellular M2 helix of the KcsA K^+^ channel (Doyle et al., 1998) as well as the M3 pore lining helix of the ionotropic glutamate receptors (GluRs) (Wollmuth and Sobolevsky, 2004) may play a role in stabilizing the closed conformation of the channel. In this case the patch will exclude water and hold the pore-lining helices of the transmembrane region together via a hydrophobic effect. In other words, a hydrophobic patch will work as a sticky patch that holds together the closed conformation of the channel in case where it is surrounded by a *hydrophilic* environment (e.g., sits at the edge of the membrane or is surrounded by polar head-groups and water). It is then the energetics of these protein–water interactions within the patch, polar lipid head-groups, and membrane-associated water that describe the stabilization/destabilization of a pore’s structural conformation. In order to assess plausibility of such hypothesis, a quantitative estimate of energetic contribution of the hydrophobic effect to the stability of a tetrameric helix bundle in hydrophilic environment of the lipid head-groups and water is needed. In this work we present such calculations using the KcsA structure (Doyle et al., 1998) to be the model system. KcsA shares structural similarity with other potassium channels that exhibit α-helix bundle crossing in a closed channel conformation, as well as with the family of GluRs. All of these proteins have a strongly conserved sequence in the region of interest for this study. The significance of this region was first discovered in the GluR delta 2 subtype. The delta 2 GluR, 25% homologous to the α-amino-3-hydroxy-5-methyl-4-isoxazolepropionic (AMPA) subtype GluR receptors (Taverna et al., 2000), are non-functional with the exception of receptors containing a spontaneous A–T mutation at the eighth position of the pore lining M3 hydrophobic patch that invokes constitutive open channels in mice (Zuo et al., 1997). These mice, known as *lurcher* mice, develop cognitive phenotypes reflecting receptor dysfunction due to a loss of neural synapses as a consequence of unregulated glutamate activity (Zuo et al., 1997; Taverna et al., 2000). Several other positions within the pore lining M3 hydrophobic patch have been found to produce *lurcher*-like channel characteristics (Kohda et al., 2000; Williams et al., 2003; Klein and Howe, 2004; Hu and Zheng, 2005; Yuan et al., 2005; Schmid and Hollmann, 2007). Constitutive activity linked to the bundle crossing region of K^+^ channels has also been identified in KcsA (Irizarry et al., 2002; Blunck et al., 2006) and voltage activated Shaker (Hackos et al., 2002; Sukhareva et al., 2003). The proximity of the helical bundle crossing in addition to the occurrence of spontaneous activation in bundle mutations in both potassium channels and GluRs are indicators of involvement of this region of the transmembrane in the gating process. Understanding this process will aid in cutting edge research targeting this region for stem cell neurotransplantation in preclinical studies of neurodegeneration in mice (Houdek et al., 2012). KcsA is chosen as a model system because it has been studied intimately. The massive amounts of experimental data as well as a number of high-resolution crystal structures available serve well for building models as well as theoretical comparison.

KcsA allows highly specific influx of K^+^ into a cell as extracellular pH decreases. It has been hypothesized that the pH sensor, located in the intracellular region of the pore lining helices, regulates the gating process for KscA (Thompson et al., 2008; Cuello et al., 2010a,b). However, neither ion selectivity nor pH sensitivity per se is the focus of the following study but rather energetics of the opening of the channel at the helix-bundle crossing due to presence of a number of hydrophobic residues lining its pore in that region. The purpose of this paper is to use the simplicity and what is known of the KcsA channel to study the effects of charge and hydrophobicity on the conformational states of a model tetrameric biological ion channel. We place focus on two important questions: (1) what force(s) stabilize the closed non-conducting channel conformation? And (2) what is the free energy associated with transitioning from a closed (water – excluding) to an open (water-filled) pore conformation?

Free energies calculated through all-atom MD simulations with enhanced sampling methods allow a quantitative characterization of the states of the model system. This data is analyzed in conjunction with the previous work of Nimigean and coworkers that provides the functional electrophysiology (Thompson et al., 2008), which together shed light onto the nature of channel conformational transitions. We suggest a similar gating mechanism may play a role for a number of ion channels containing a hydrophobic bundle crossing in proximity to the water–lipid interface of a membrane.

## Materials and Methods

### Model Systems

**FIGURE 1:**
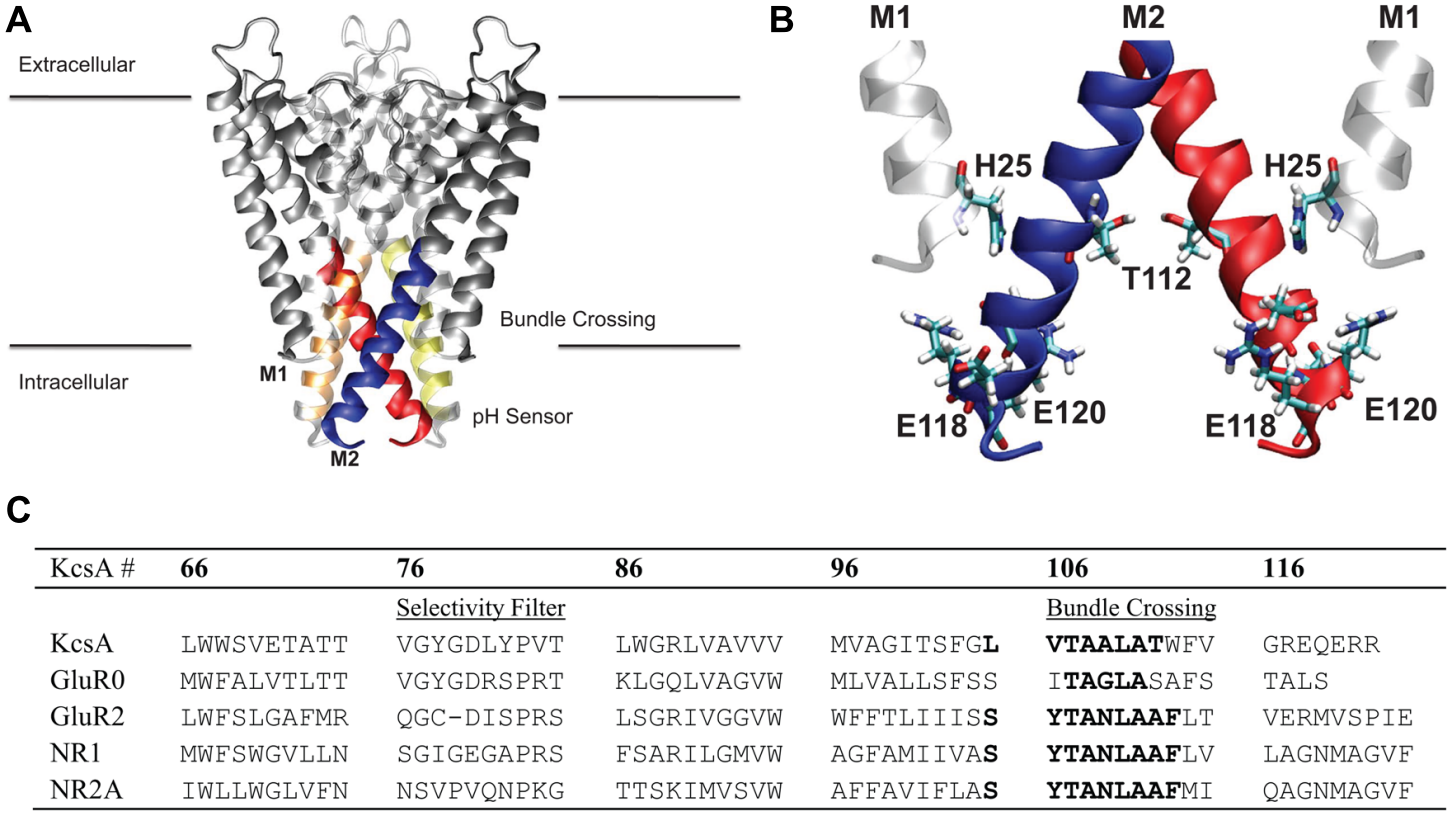
**Membrane region of the KcsA channel structure and sequence. (A)** Protein is shown in gray ribbon representation. Regions colored blue, red, orange, and yellow correspond to the stretch of residues within the hydrophobic patch of the helical bundle crossing discussed in this study. **(B)** Key residues involved in the pH sensor of KcsA are indicated in CPK coloring and bonds representation. The M1 residue, H25, involved in the pH sensing machinery was mimicked in the model using the adjacent M2 helix T112 residue as indicated. **(C)** Sequence alignment of the KcsA region of interest and four different subunits from the glutamate receptor (GluR) family of ion channels AMPA (GluR0 and GluR2) and NMDA (NR1 and NR2A). Sequence numbering corresponds to KcsA residue positions.

The structure of the KcsA K^+^ channel from *Streptomyces lividans* solved to 2.0 Å resolution using X-ray crystallography in a closed channel pore conformation (PDB ID: 1K4C) (Zhou et al., 2001) was used as the starting point for calculations. The open channel pore conformation was calculated using a homology model threading procedure of the KcsA sequence onto the 3.3 Å resolved crystal structure of MthK (PDB ID: 1LNQ) in an open conformation using MODELER (Sali and Blundell, 1993). Although protein–protein and protein–lipid interactions should be taken into consideration (White and Wimley, 1999), a simplistic model of the hydrophobic effect on the channel pore is our goal and subsequent studies have included full protein and membrane systems (see Discussion). Therefore, this study includes only residues T85 to H124 (M2 in KcsA language) in our model, The resulting protein consisted of four helices; one M2 from each of the four subunits and is referred from here on as the M2 helical bundle as shown in **Figure 1A**. **Table 1** shows the several mutant models simulated all based on the wild-type helical bundle described. All MD simulation systems consisted of protein solvated in TIP3P water, they were subject to 2 · 10^4^ steps of conjugate gradient energy minimization with protein atoms restrained using a harmonic restraint of 50 kcal/mol/Å^2^. Next they were simulated in the NVT ensemble in a 250 ps step-wise procedure to slowly equilibrate the system over a 1 ns time period. Subsequent MD simulations were performed in the NPT ensemble at 300 K in AMBER 10 (Case et al., 2008) utilizing the Cornell et al. (1995) force field. Temperature was controlled with the Berendsen thermostat (Berendsen et al., 1984) and bond lengths involving hydrogen atoms were controlled with the SHAKE algorithm (Ryckaert et al., 1977) as dynamics were collected at 2 fs/step.

**Table 1:** Model systems^∗^.

Model	Description	Model type
(1) Wild-type	Wild-type M2 bundle (T85–H124)	Native
(2) E118A/E120A	Computational double mutant^∗∗^	Hydrophobic
(3) E118A/E120A/T112^+^	Computational double mutant and charge modulation	Hydrophobic and electrostatic
(4) A108S/A111S	Computational double mutant	Hydrophilic
(5) A108S/A111S/V115S	Computational triple mutant	Hydrophilic
(6) E118A/E120A/R121^+0.5^R122^+0.5^	Computational double mutant and charge modulation	Hydrophobic And electrostatic
(7) Wild-type non-polar	Protein in non-polar solvent	Hydrophobic
(8) Wild-type patch	Small hydrophobic bundle only (L105–T112)	Hydrophobic

*^∗^Model systems were all prepared under the same simulation conditions as described in the methods section.*

*^∗∗^Computational mutations were imposed and the resulting model was re-equilibrated as described in the methods section.*

Umbrella sampling (US), a well-known enhanced sampling technique used to compute a potential of mean force (PMF) between two known states of a system along a predefined transition path (Chipot and Pohorille, 2007) was utilized here to model free energies of bundle opening. US is a method to impose biasing potentials (termed umbrellas) to confine sampling of a simulated system to a subspace of its conformational space thus enhancing the sampling of rarely visited conformations. Here we used two-dimensional US with harmonic biasing potentials applied to helices positioned in the tetramer bundle. Umbrellas were placed on the distance between two single C_α_ atoms for each pair of G116 residues diagonally across the channel pore from each other. Each US window was simulated using a two-dimensional quadratic harmonic potential of the form

(1) $$V(\zeta_{1}\;\zeta_{2})=\frac{1}{2}[k_{1}{\left(\zeta_{1}-\;\zeta_{1}^{0}\right)}^{2}+k_{2}{\left(\zeta_{2}-\;\zeta_{2}^{0}\right)}^{2}]$$

where ζ_1_ and ζ_2_ are the two reaction coordinates for the system, $\zeta_{1}^{0}$ and $\zeta_{2}^{0}$ are the equilibrium distances of the respective reaction coordinate in a particular window, k_1_ and k_2_ are the harmonic force constants that have an equivalent value of 3 kcal/mol/Å^2^. This potential was then unbiased from the resulting simulations using the Weighted Histogram Analysis Method (WHAM; Grossfield, 2010). Convergence was found after a maximum of 30 iterations with no need for special initial free energy values. Subsequent unbiasing calculations resulted in identical free energy values. Sampling the entire conformational space of the helical bundle using two-dimensional umbrellas at every 0.5 Å would require 144 MD simulations per system. Since the bundle is tetrameric and our reaction coordinates are symmetric the space can be reduced by half to 72 simulations per system.

Initial symmetric structures were constructed at every 0.5 Å along the reaction coordinate using the FRODA module of the FIRST flexibility analysis program (Thorpe et al., 2001). Asymmetric structures were then generated from symmetric ones manually using the HARLEM software package (Kurnikov, 1996). A single exhaustive calculation of 72 independently equilibrated MD simulations revealed the conformational energy surface to also be radially symmetric about the minimum or maximum value of the free energy. Symmetry was exploited to allow for fewer umbrella simulations in the subsequent simulation, and only 10–12 umbrellas were used along the diagonal of the free energy surface. Convergence was monitored using WHAM convergence criterion by increasing simulation time in increments of 1 ns. Non-equilibrium portions of the trajectory (ca. 500 ps–1 ns) were removed from the total simulation for a total of 4–5 ns long dataset of equilibrated production runs. The one-dimensional PMF profiles shown in all the figures presented here represent 40–50 ns of total simulation time, and two-dimensional PMF profiles represent 2.9–3.6 μs of total simulation time.

### Non-polar Solvent Model

A simple non-polar solvent was constructed using standard single point charge (SPC) water molecules with modified Leonard–Jones parameters and dipole moment. The well-depth Leonard–Jones parameter from the aliphatic carbon atoms within a reduced model for a dimyristoyl phosphatidylcholine (DMPC) lipid molecule (Matyus et al., 2006) were used in place of the standard water parameters. This substitution changes the depth of the pair-potential well for the solvent molecule. In addition, the overall dipole moment of water was reduced by half to mimic a non-polar molecule.

### Water Density Calculations

Water densities were calculated in the confined region of the M2 bundle between residue L105 and H124 as the average number of water molecules per cubic angstrom. For each equilibrium umbrella simulation the average number of waters was calculated and plotted as a function of the channel axis (z). The radius of the channel at residue T112 with a channel axis slice of 1 Å thick determined the volume of the calculation box. In this formalism a value of 0.003345 molecules/Å^3^ corresponds to 1.3 g/ml, the bulk density of water.

## Results

### Conformational Free Energy Landscape of KcsA M2 Helical Bundle

In this work we report on the energetics of open to closed transition of a truncated tetrameric helical bundle that is formed at the lipid/water interface in similar fashion in a number of tetrameric ion channels. The results presented here are simulated using the wild type sequence and initial structure of the KcsA K^+^ channel from *mus musculus* in a closed channel pore conformation (PDB ID: 1K4C) (Zhou et al., 2001), as well as a number of mutants of this channel. Only the effect of the sequence and structure forming this bundle closing is assessed in this work. Other factors, such as nearby hydrogen bonds, the presence of the rest of the structure and the lipid bilayer were not included in the simulations. Potentials of mean force were calculated for the conformational transition from a closed M2 helical bundle to an open M2 helical bundle using a two-dimensional reaction coordinate describing the distance between two opposite helices as described in Methods section.

**FIGURE 2:**
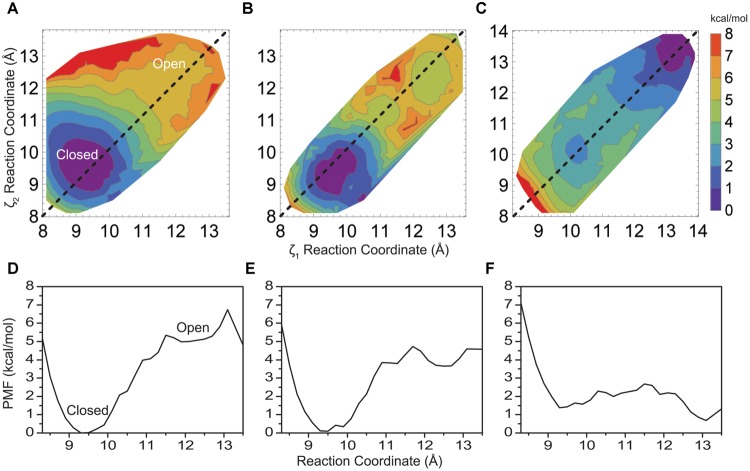
**(A)** Two-dimensional PMF profile for native KcsA M2 bundle, **(B)** mutant E118A/E120, and **(C)** mutant E118A/E120A/T112^+^ KcsA M2 bundle shown in a contour plot as a function of reaction coordinates ζ_1_ and ζ_2_. Each colored contour represents approximately 1 kcal/mol. **(D)** One-dimensional profile (ζ_1_ = ζ_2_) of the PMF for the native KcsA M2 bundle, **(E)** mutant E118A/E120A, and **(F)** mutant E118A/E120A/T112^+^ KcsA M2 bundle shown in **(A–C)** as dotted lines.

The PMF for opening of the helix bundle is shown in **Figure 2A** in a contour plot as a function of reaction coordinates ζ_1_ and ζ_2_ (Eq. 1), where each colored contour is approximately 1 kcal/mol. The two Cartesian reaction coordinates ζ_1_ and ζ_2_ are defined by the distance between opposing helices when drawn through the rotationally symmetric axis of the pore. [Note, we define an “open” conformation here as a structure where water consistently resides inside and throughout the entire length of the pore. This is done to strictly limit simulation time and calculate energies, as the actual biological open state of the pore may be different]. The PMF reveals an energetic difference of 5.3 kcal/mol between states for the native M2 bundle at (ζ_1_, ζ_2_) = (11.5, 11.5) Å and maintains a shallow minimum around 5 kcal/mol as the reaction coordinate (bundle opens) increases. A well-defined energy minimum at (9.4, 9.6) Å is resolved from the simulations and occurs close to an inter-helical distance defined by the crystallographic starting structure of closed KcsA at (9.6, 9.6) Å. All unconstrained simulations started within the region of a reaction coordinate distance of (8, 8) – (10, 10) Å reverted back to an inter-helical distance of 9.6 Å observed in the PMF of **Figure 2A** similar to the crystal structure of closed KcsA. A tightly formed M2 helical bundle is formed during non-restrained simulations within 2–4 ns (results not shown). This observation validates the energy minimum around (9.4, 9.6) Å as seen in the PMF of **Figure 2A** and we define this conformation as a stable closed conformation of the native M2 helical bundle.

The PMF profile in **Figure 2A** was computed using 144 2-d umbrella windows. Close inspection reveals its symmetric nature with respect to the symmetric opening of the bundle (dashed line showing ζ_1_ = ζ_2_ in **Figure 2A**), as well as monotonic and sharp increase in energy along all directions normal to the line of symmetric opening. Thus the amount of the simulations required to obtain the energy difference between the open and the closed state of the helix bundle, and an estimate of a reaction coordinate and barrier height for this transition, may be drastically reduced by simulating only 72 umbrella windows. To simplify presentation and discussion it is also instructive to have a one-dimensional plot along the line ζ_1_ = ζ_2_, as shown in **Figure 2D** extracted from **Figure 2A**. In the following, all 2-d PMFs profiles shown are made with 12 umbrella windows and the corresponding 1-d plots are shown to simplify the presentation and inspections of the presented PMFs.

To gain insight into the forces that provide about 5 kcal/mol energy barrier between a closed helix bundle and alternative conformations, mutations previously reported experimentally, were introduced into the helix bundle model computationally. Following the experiments of Thompson et al. (Thompson et al., 2008) we introduce E118A and E120A residue mutations to the helix bundle. These residues are located in the highly charged region of the intracellular KcsA M2 bundle crossing (**Figure 1**) and are suggested to be part of the KcsA pH sensor (Cortes et al., 2001; Takeuchi et al., 2007). Upon mutation, experimental single channel recordings indicate a flickering of the channel from open to close state up to pH 6.5 suggesting a disruption of the M2 pore equilibrium by altering the open channel probability slightly in favor of the open conformation (Thompson et al., 2008). Indeed, PMF calculations shown in **Figure 2B** reveal a 4.1 kcal/mol barrier at 11.5 Å when transitioning to the open conformation of the simulated helix bundle with the E118A/E120A mutation consistent with this observation.

Electrophysiological experiments of mutant KcsAs performed by Thompson et al. (2008) revealed that the pH dependence of channel opening is removed only when H25, shown in **Figure 1** as located on M1 and proximal to the hydrophobic patch, is protonated or mutated to contain a positive charge (Takeuchi et al., 2007). To mimic the effect of protonation of mutation of H25 in the model, an extra positive charge was assigned on the adjacent T112 residue to contain either a +0.5e or a +1.0e charge. This technique has been used successfully in other simulation systems as an alternative to the intensive process of thermodynamic integration (Mamonova et al., 2008). Introducing a charge into the hydrophobic patch alone via the T112^+1^ charge modulation drastically changes equilibrium (relative energy difference) between the closed and open conformations of the M2 bundle. **Figure 2C** shows that the positive charge modulation favors the open conformations of the M2 bundle. A barrier of 3 kcal/mol at (11.5, 11.5) Å separates the closed and open conformations of the M2 bundle. When the E118A, E120A along with the T112^0.5+^ charge mutations are introduced the energetic barrier at 11.5 Å is reduced as shown in the free energy projection plot of **Figure 2F**. This distribution of free energy states is synonymous with an M2 bundle in which all states of open and closed conformation are equally probable. **Figure 2F** shows the bundle favors the open conformation by only 0.5 kcal/mol. The probability is completely shifted to the open conformation of the M2 bundle by introducing the more severe T112^1+^ perturbation. As **Figure 2** shows, the addition of two hydrophobic residues and a small addition of charge change the PMF profile significantly. No barrier exists for the closed to open M2 bundle conformational transition in this case (**Figure 2F**).

The intracellular region of KcsA is organized as shown in **Figure 1**. Polar residues that allow favorable inter-helical hydrogen bonding to create the geometry of the channel flank the hydrophobic patch. Separate from the bundle crossing is a stretch of charged residues creating a complicated network of hydrogen bonds and charge interactions. The energetic interactions observed among residues within intracellular M2 and the bundle crossing can thus be separated into two classes: hydrophobic and electrostatic interactions.

### Effects of Pore Hydrophobicity on the Free Energy Profile of the M2 Bundle

**FIGURE 3:**
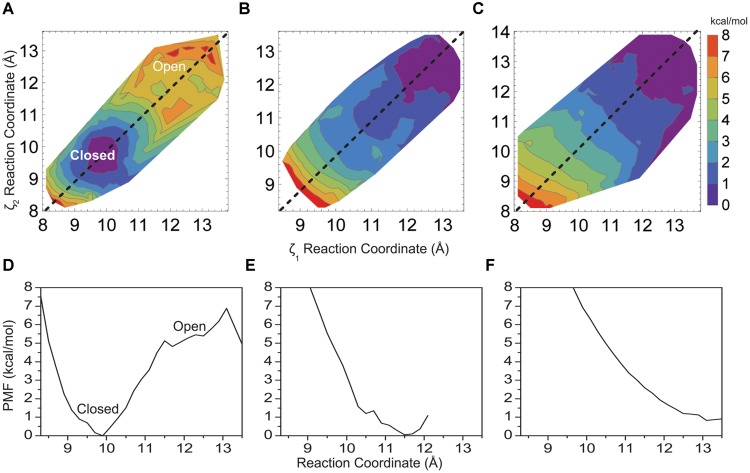
**Two-dimensional PMF for the KcsA M2 bundle hydrophobic **(A)** double mutant A108S/A111S, **(B)** triple mutant A108S/A111S/V115S, and **(C)** the native KcsA M2 bundle in the presence of a model non-polar solvent as a function of reaction coordinate.** One-dimensional profile (ζ_1_ = ζ_2_) of the PMF for **(D)** double mutant A108S/A111S, **(E)** triple mutant A108S/A111S/V115S, and **(F)** KcsA in the presence of a model non-polar solvent.

**Figure 3** shows how changing the hydrophobicity of the pore lining residues affects the free energy barrier between a closed and open helix bundle conformation. Changing hydrophobic pore lining residues A108 and A111 to polar serine had only a slight effect on bundle stability (**Figures 3A,D**), as the barrier remains similar to the 5 kcal/mol native M2 barrier. A shift of the PMF minimum to ca. 10.0 Å for the more polar M2 bundle indicates a still stable closed bundle, however, the pore diameter is slightly wider than with the native sequence and pore hydration calculations similar to those shown in **Figure 5A** show that pore solvation is slightly increased. The next amino acid to face the pore (i+4) is V115. Single substitutions of V115S yields results similar to A108S and A111S, that is little disruption of the 5 kcal/mol barrier (data not shown). Although, adding the third sequential hydrophilic substitution in the pore lining, V115S, to the A108S and A111S mutations sharply disrupted the stability of the closed helix bundle conformation. **Figures 3B,E** shows a single PMF minimum for a triple mutant around the wide-open 12.5 Å M2 bundle.

To further validate the idea of hydrophobic interactions defining the free energy difference between closed and open conformations of the native helix bundle, a non-polar solvent model was generated using SPC water by adjusting the well depth of the water Lenard–Jones potential and then lowering the water dipole (as described in Methods). **Figures 3C,F** show how the PMF of the native M2 bundle changes when solvated with the model non-polar solvent (model 7, **Table 1**). Importantly, strikingly similar to the mutant model where hydrophilic residues line the pore (model 5, **Table 1**) as shown in **Figures 3B,E**. For both cases the barrier between closed and open conformations of M2 is reduced to zero.

### Free Energy Profile for a Four Helix Hydrophobic Bundle

**FIGURE 4:**
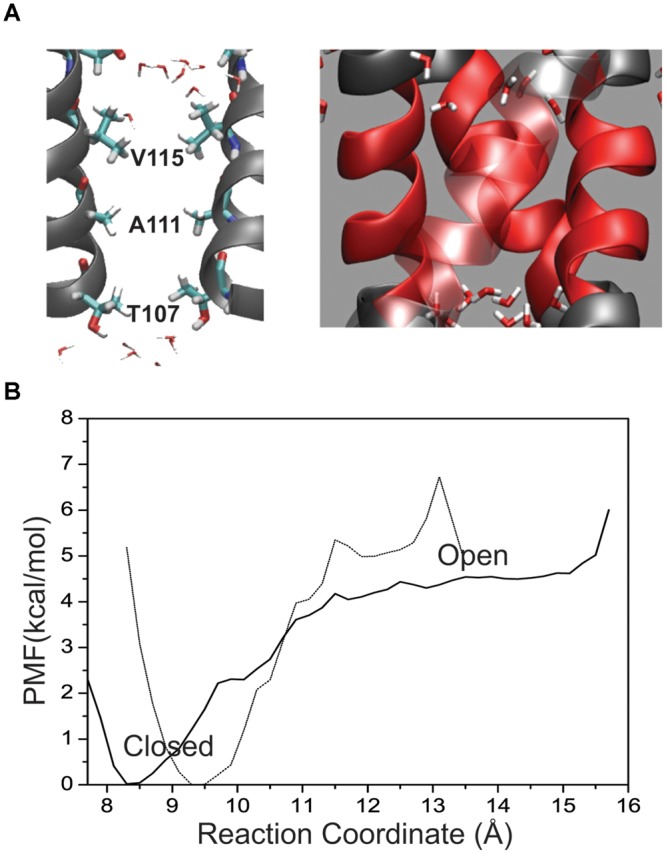
**(A)** Placement of residues within the KcsA M2 bundle crossing where two helices have been removed for visual clarity. Pore residues are shown in a bonds representation and CPK coloring. Water molecules are shown at the top and bottom of the patch as they are excluded from the patch in the channel-closed conformation. **(B)** One-dimensional profile (ζ_1_ = ζ_2_) of the PMF for the hydrophobic patch only (64 residues) in the KcsA M2 bundle (solid line) shown together with the full wild-type bundle crossing (dashed line).

To obtain an estimate of free energy contributed solely by the hydrophobic patch the M2 helical bundle was further truncated to the region between L105 and T112 as shown in **Figure 4A**. **Figure 4B** shows the PMF for this small hydrophobic bundle. For the native M2 sequence a barrier of approximately 4 kcal/mol arises as the inter-helical distance increases. The minimum of the PMF for this small bundle can be seen as shift to 8.4 Å and reflects the closer association of the helical bundle in the absence of the surrounding structure of M2 and KcsA protein. A more hydrophilic M2 bundle was created with the same polar computational mutation made in the larger M2 bundle of A108S/A111S. The PMF for the polar M2 bundle (not shown) with the hydrophobic patch alone reveals a complete reduction of the barrier. The full mutant as described earlier (i.e., E118A/E120A/T112^+^) is obviously not possible for this truncated system as the intracellular region was removed. Interestingly, when the single T112^+^ charge mutation was incorporated into the truncated M2 hydrophobic patch, all but the largest diameter umbrella simulations became unstable indicating a huge disruption of the M2 patch due to the local increase in charge.

### Wetting and De-wetting of the Helix Bundle Pore

**FIGURE 5:**
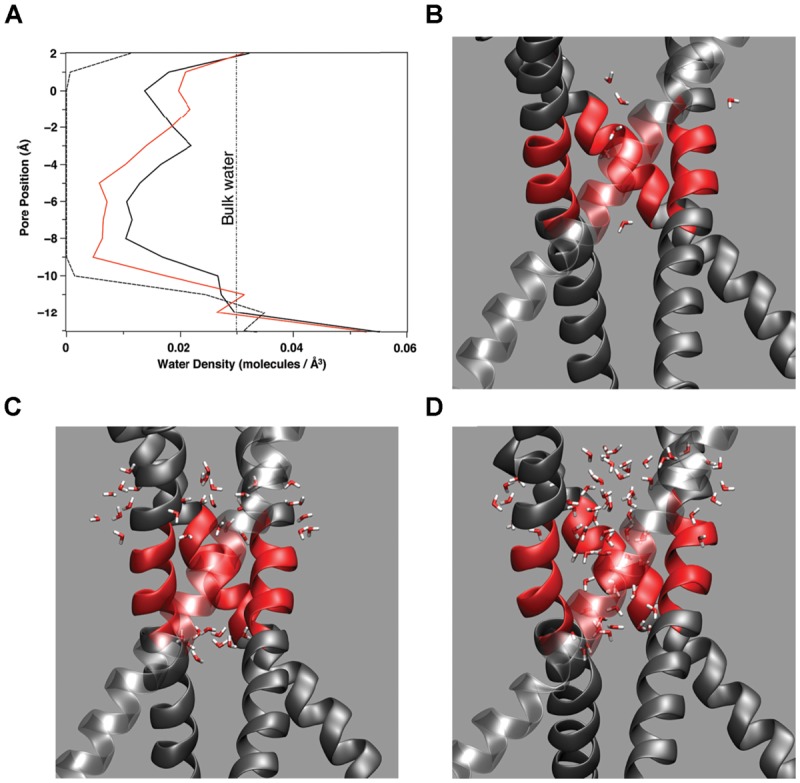
**(A)** Water density as a function of location along the (*z*) pore axis for three amino acid mutations: A108S/A111S (dashed line), E118A/E120A/T112^+^ (red line), A108S/A111S/V115S (black line). The curves are constructed from the average number of water molecules within a 1 Å thick slice of the pore over 2 ns simulation time during stable, fully equilibrated simulations. The vertical dashed line indicates the average bulk density of water. Partial snapshots from simulations with the hydrophobic patch of M2 highlighted in red are shown for a **(B)** dewetted patch, **(C)** partially wetted pore region, and **(D)** fully hydrated pore region.

Water presence within the pore changes as a function of pore radius and amino acid makeup of the pore lining. **Figure 5A** plots the water density within the pore as a function of distance along the pore (z) axis for several amino acid mutations. In simulations where the pore reached between 10.5 and 11.0 Å at the top of the hydrophobic patch (T112 Ca–Ca distance), water density begins to fluctuate. Note that the changes in water density at this region of the pore are not linear with subsequent increasing pore size. **Figure 5** reveals the outermost regions of the hydrophobic patch undergo wetting-de-wetting transitions as indicated by a drop in water density in the regions of T112 (*z* = -0.5 Å) and L105 (*z* = -10.0 Å).

## Discussion

Cuello et al. (2010a,b) have produced an open conformation of KcsA using a very similar set of mutations as outlined in this work and our structures are in agreement with their crystal structures to 1.0 Å RMSD. Defining an open conformation that is ultimately described and discussed without knowledge of an open structure is a dangerous approach, although identifying a characteristic common to a family is helpful in inferring structure and function of family members. Most K^+^ channels function to conduct ions across the pore controlled by some type of gate that occludes the pore until the conditions are right for ion passage. Ion entry into the channel pore is not a single-ion procedure as the ion must be solvated or protein coordinated according to its ionic charge. It is thus assumed that a channel can conduct current when a minimum radius allowing water entry to the pore is maintained. With this in mind, ensembles of open KcsA M2 bundle conformations generated by the FRODA framework rigidity calculations between the closed crystal structure and an open homology model of KcsA provided varying degrees of channel solvation. The final model defined in this work as an “open” state or conformation of KcsA was determined based on a “just wetted” channel pore during MD simulations. Equilibrated models were examined for the transition from a de-wetted (vacuum bubble) to a wetted (hydrated) pore within the region of the bundle-crossing hydrophobic patch. Comparing the inferred open conformation of the KcsA M2 bundle to the full-length open crystal structure of KcsA reveals high similarity with Cα RMSD within 1.0 Å (Cuello et al., 2010b). Thus these new crystal structures provide strong support for the herein definition of an open conformation of the M2 bundle and show biological significance for the subsequent free energy comparison between a closed and open equilibrium pore conformation.

Free energy calculations were used to answer a number of outstanding questions concerning the effects of hydrophobicity and charge involved in ion channel conformational changes. Clearly these effects are present in KcsA, which is not to say these are the *only* forces that govern KcsA conformational changes. We use KcsA as a model to quantify energy differences between equilibrium states of the M2 bundle. Based on the hypothesis that hydrophobicity of residues creating the inner surface of the channel pore constitutes a hydrophobic gate for ion conduction, a quantitative analysis of the M2 patch within KcsA was conducted. The amino acids within the bundle crossing provide a significant contribution to the conformational free energy profile of the KcsA M2 bundle as shown in **Figures 2** and **3**. Disrupting these favorable interactions requires energy to allow ion entry into the pore. Hydrogen bonding and hydrophobic interactions in the intracellular region of KcsA stabilize the pore lining M2 helices at physiologic pH. Reducing intracellular pH provides the energy to disrupt the M2 patch and increase the probability of an open conformation of the M2 bundle.

### Hydrophobicity as a Gating Mechanism

Membrane proteins require a large ratio of non-polar to polar residues in order for insertion and functional viability with the low dielectric of the biological membrane (Hille and Catterall, 1999). Many membrane proteins contain an interruption within channel spanning pores by a short stretch of hydrophobic residues anywhere from three (as in the KcsA channel) to six residues long (as in the acetylcholine receptor) (Miyazawa et al., 2003). A hydrophobic patch within a water pore can be thought of as a “sticky” point on the channel pore-lining surface which when associated with a neighboring surface excludes water to the boundary of the patch. Changing the composition of this protein surface to a more polar character will alter its ability to interact favorably with its partner and solvating that surface with water is more favorable. This phenomenon has previously been observed in MD simulations of biological channels (Hummer et al., 2001; Beckstein and Sansom, 2003, 2006; Anishkin and Sukharev, 2004; Liu et al., 2005; Jensen et al., 2010).

In the absence of surrounding charges or other protein structure **Figure 4** shows a free energy barrier due to a pure biological hydrophobic bundle (L105–T112). However, imposing experimentally relevant perturbations to such a small system (64 amino acids) causes instabilities in the simulations and unreliable comparisons. The results summarized in **Figure 3** clearly show that modulating the ability for residues within the patch to interact with water can also alter the conformation of a larger KcsA M2 bundle, which includes the charged intracellular region. This shift in probability from a mostly associated bundle (closed state) to a mostly dissociated bundle (open state) is controlled at the amino acid side chain level. A more polar side chain, (i.e., serine versus alanine) creates a surface where the volume forces of water association with itself are greater than the water-surface association properties of protein–water interaction. This phenomenon has been observed in reduced models of KcsA that incorporate only geometrical properties of the channel (no electrostatics) (Roth et al., 2008). As the radius of the M2 bundle increases density fluctuations in water at the boundaries of the hydrophobic patch give rise to a water phase transition. The vacuum created by the hydrophobic surface of the M2 bundle association becomes wetted with a water droplet. When the radius of the channel is large enough, the volume forces of water become larger than the hydrophobic forces between M2 helices resulting in a fully hydrated pore and an open M2 bundle. The result of **Figure 3** is significant in that it provides evidence for a hydrophobic gate controlled by modulating surface characteristics of the pore lining residues of a protein.

### Gating in Channels Containing a Hydrophobic Bundle Crossing Located at a Membrane–Water Interface

If the mechanism of allowing or inhibiting ion conduction through a channel containing some form of hydrophobic bundle crossing is a balance between water–water and water–protein interactions, then the positioning of the channel within the lipid bilayer becomes important. The closed, non-conducting, state of the channel pore is solvated with water along the length of the pore except for regions of increased hydrophobicity (Hummer et al., 2001; Beckstein and Sansom, 2003, 2006; Anishkin and Sukharev, 2004; Liu et al., 2005; Jensen et al., 2010). Water density calculations in the region of the hydrophobic patch of KcsA shown in **Figure 5**, as well as simulations of full length Kir1.2 in a membrane (Jensen et al., 2010) show the water density in these regions of the pore are below the bulk density of water (1.0 g/ml or 0.0034 molecules/Å^3^). The mechanism for disrupting a hydrophobic patch as described in this discussion will require a nearby water source with bulk properties able to induce the vapor to water phase transition within the region of the bundle hydrophobic patch. The interface provided by the lipid head groups of the membrane and either the cytosol or extracellular fluid could provide such an environment for favorable hydrophobic gating in channels.

As a test of generality for the proposed mechanism of hydrophobic gating, consider the ligand-gated AMPA and NMDA subtypes of the ionotropic GluRs. **Figure 1** shows a sequence alignment of AMPA GluR2 and NMDA NR1/NR2A sequences with KcsA. Inspection of the bundle-crossing regions of these channels reveals a conserved sequence: SYTANLAAF. This sequence forms a bundle-crossing region in the full length tetrameric X-ray crystallographic structure of GuR2 similar to structures of K^+^ channels (Sobolevsky et al., 2009). The existence of a homologous bundle-crossing regions formed by a stretch of hydrophobic residues that exhibit conformational dependence due to an external stimulus suggests a possible channel gating mechanism. A perfectly homologous patch between channel proteins is not required and should not be expected. The asparagine residue that interrupts the hydrophobic bundle crossing of GluRs uniquely faces the ion channel pore and has been shown to infer Ca^2+^ selectivity in AMPA receptors (Jatzke et al., 2003). The bundle crossing lies on the extracellular side of the membrane for GluRs, where the membrane region transitions to a ligand-binding region. The lack of homology between the highly charged intracellular region of KcsA and the corresponding extracellular region of GluRs reflects the differences in channel activation for these channels. GluRs are ligand-gated and, for the case of the NMDA receptor subtype, voltage gated and consequently do not rely on changes in local electrostatics to drive channel opening. The energy for disruption of the hydrophobic bundle crossing within GluRs would primarily come from ligand binding to the extracellular domain. Conformational transitions within the LBD are transferred to the transmembrane region through short peptides, providing the energy required to disrupt the bundle crossing in the form of mechanical force. Theoretically, removal of the LBD would cause the transmembrane region to be found mostly in a closed channel conformation. Only by disruption of the hydrophobic bundle crossing with polar mutations would the ion channel be found with high probability in a conducting state or open conformation. Simulations of the transmembrane regions for both AMPAR and NMDAR are currently being conducted to validate these hypotheses quantitatively.

## Conflict of Interest Statement

The authors declare that the research was conducted in the absence of any commercial or financial relationships that could be construed as a potential conflict of interest.
